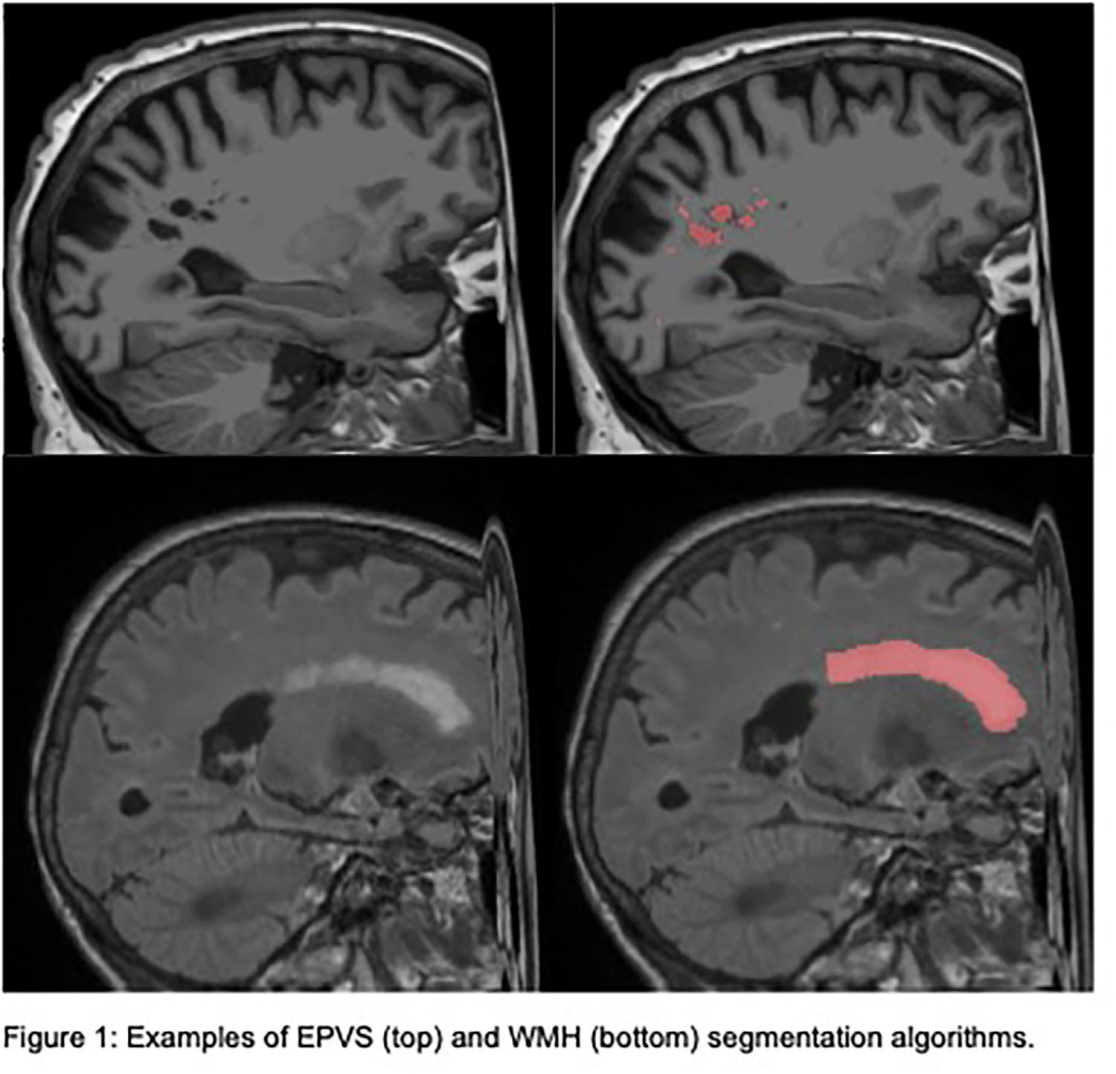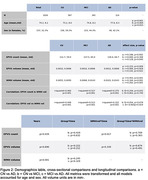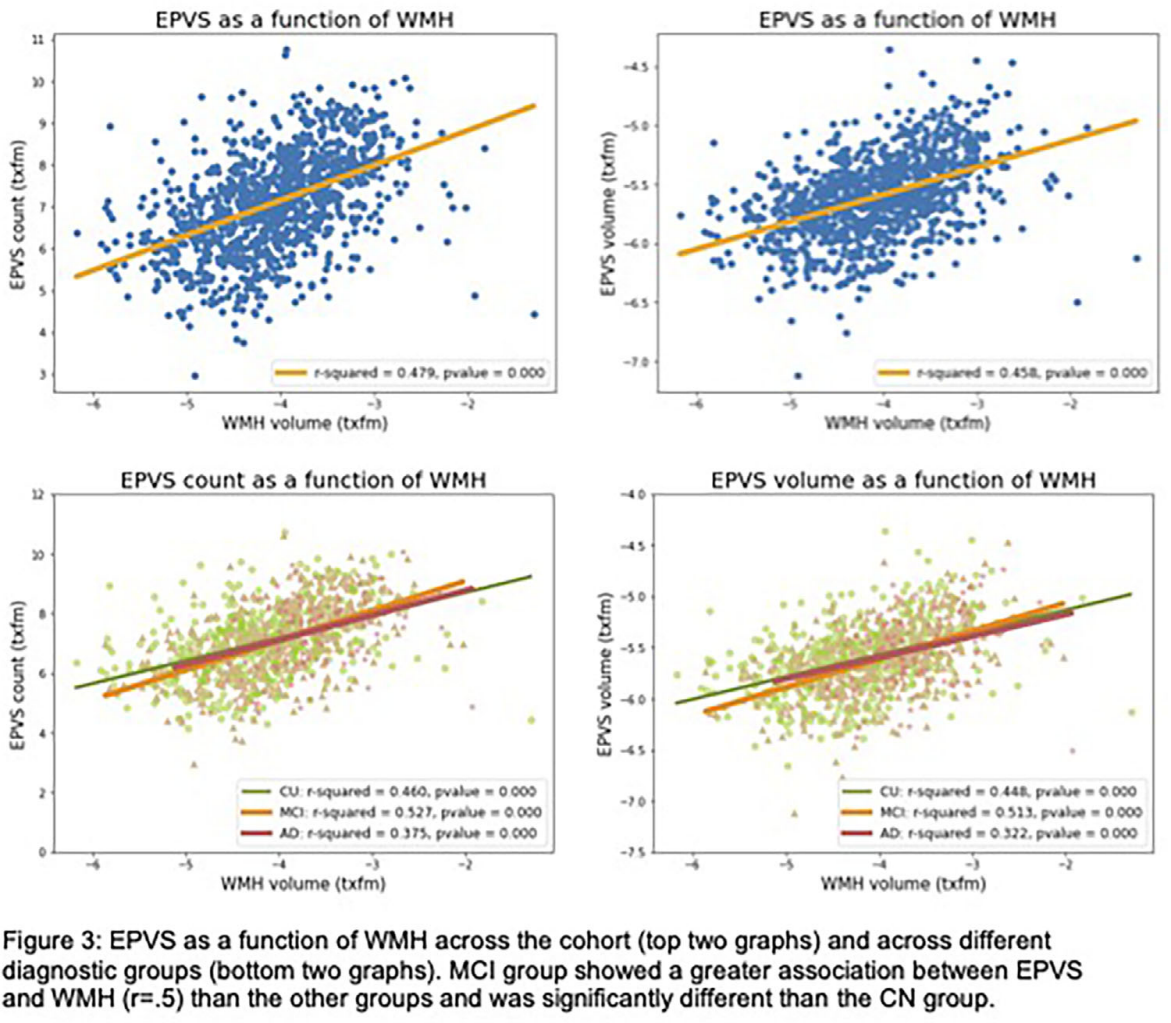# Relationship between perivascular spaces and white matter hyperintensities in Alzheimer’s disease

**DOI:** 10.1002/alz.094806

**Published:** 2025-01-09

**Authors:** Serena Tang, Duygu Tosun

**Affiliations:** ^1^ University of California ‐ San Francisco, San Francisco, CA USA; ^2^ University of California ‐ Berkeley, Berkeley, CA USA; ^3^ Department of Veterans Affairs Medical Center, Northern California Institute for Research and Education (NCIRE), San Francisco, CA USA; ^4^ University of California, San Francisco, San Francisco, CA USA; ^5^ Department of Radiology and Biomedical Imaging, University of California, San Francisco, San Francisco, CA USA; ^6^ Center for Imaging of Neurodegenerative Diseases, San Francisco Veterans Affairs Medical Center, San Francisco, CA USA; ^7^ San Francisco Veterans Affairs Medical Center, San Francisco, CA USA

## Abstract

**Background:**

There is increasing evidence that cerebrovascular and glymphatic‐related deficits may precede and influence AD pathology. In this study, we investigated the relationship between enlarged perivascular spaces (EPVS) as a measure of glymphatic clearance integrity and white matter hyperintensities (WMH) as a measure of cerebrovascular integrity.

**Method:**

Participants from ADNI‐3 (N = 1026) who were diagnosed as cognitively unimpaired (CU) (n = 567), mild cognitively impaired (MCI) (n = 345), or AD dementia (n = 114) were included (Figure 2). Up to five visits (approximately one visit per year) were recorded for each participant. EPVS were segmented and quantified on 3T T1‐weighted images using a novel fully‐automated algorithm, while WMH were segmented on FLAIR images using the Lesion Segmentation Toolbox (Figure 1). Pairwise comparisons between EPVS and WMH metrics were tested using Mann‐Whitney U‐tests. A GLM was used to model the relationship between EPVS and WMH in each diagnostic group. LMMs were used to determine whether changes in WMH influenced EPVS changes between the groups. Age and sex were included as potential confounding factors, and data were transformed as needed.

**Result:**

In the cross‐sectional analysis, the AD group had more EPVS count, volume, and WMH volume than the CU and MCI groups (Figure 2). A significant association was found between EPVS metrics and WMH volume (R‐squared = 0.4; p<0.001; Figure 3). MCI compared to CU presented a stronger association between EPVS count and WMH volume (p = 0.02). In the longitudinal analysis, EPVS count and volume significantly increased with time, however, no differences were found between groups (Figure 2). Changes in EPVS count were associated with changes in WMH volume, however, this association did not significantly differ between groups (Figure 2).

**Conclusion:**

We found that the association between measures of EPVS and WMH varies with different clinical stages of AD. This suggests that cerebrovascular and glymphatic‐related deficits may interact and exacerbate in all clinical stages with varying degrees and timing. Untangling the relationship between these pathophysiological changes and their influence on AD progression will further our understanding of the etiology of AD and highlight potential modifiable risk factors.